# Behavioral Symptoms after Breast Cancer Treatment: A Biobehavioral Approach

**DOI:** 10.3390/jpm5030280

**Published:** 2015-08-03

**Authors:** Christopher Fagundes, Angie LeRoy, Maryanne Karuga

**Affiliations:** 1 Department of Psychology, Rice University, Houston, TX 77005, USA; E-Mail: christopher.fagundes@rice.edu; 2 Department of Symptoms Research, MD Anderson Cancer Center, Houston, TX 77030, USA; 3 Department of Psychiatry, Baylor College of Medicine, Houston, TX 77030, USA; 4 Department of Psychology, University of Houston, Houston, TX 77004, USA; E-Mail: asleroy@uh.edu; 5 Department of Science, Technology, Engineering, and Mathematics, Dillard University, New Orleans, LA 70122, USA; E-Mail: maryanne.karuga@dillard.edu

**Keywords:** breast cancer survivors, inflammation, SES, chronic stressors, proinflammatory cytokine production, recurrence, fatigue, depression

## Abstract

Being diagnosed and treated for breast cancer is emotionally and physically challenging. Breast cancer is the most commonly diagnosed cancer and the second leading cause of death for women in the United States. Accordingly, women with a breast cancer history are the largest group of female cancer survivors. Psychological stress substantially augments adverse autonomic, endocrine, and immune discharge, including enhanced production of proinflammatory cytokines. Importantly, inflammation is a key biological mechanism underlying the symptom cluster of pain, depression, fatigue, and sleep disturbances; there is also good evidence that inflammation contributes to breast cancer recurrence. Stress may exert direct effects on psychological and physiological risk processes. In this review, we take a biobehavioral approach to understanding predictors and mechanisms underlying somatic symptoms in breast cancer survivors.

## 1. Introduction

Being diagnosed and treated for breast cancer is emotionally and physically challenging. Breast cancer is the most commonly diagnosed cancer and the second leading cause of death for women in the United States [1]. There are more than 2.3 million breast cancer survivors in the United States, a number that is expected to dramatically increase; advances in adjuvant therapy combined with early tumor detection have dramatically improved disease-free survival for breast cancer survivors [1]. Accordingly, women with a breast cancer history are the largest group of female cancer survivors.

When primary treatment-related problems subside, many breast cancer survivors continue to report a heavy symptom burden that includes fatigue, depression, and disrupted sleep [2,3]. The survivorship period requires management of these ongoing physical symptoms and fears of recurrence. As the number of breast cancer survivors is projected to dramatically increase, more attention is being devoted to these issues [4].

In this paper, we provide a brief overview of the current state of the literature on post-treatment symptom burden in breast cancer survivors. Using Medline, CINAHL, and Psychinfo, (1960-week-present), we searched using recognizable terms in the breast cancer survivorship literature. The following words were used in combination with breast cancer survivors: symptoms, quality of life, fatigue, depression, pain, somatic, and sleep. This paper is not intended to be a systematic literature review and thus the themes presented below should not be viewed as comprehensive.

## 2. Symptom Overview

Behavioral symptoms such as fatigue, depression and sleep disturbance are among the most common and deleterious post-treatment problems in breast cancer survivors [5]. Just over a decade ago, an NIH State of the Science review concluded that post-treatment symptoms were undermanaged in cancer care and recommended an increased effort to develop evidence that would support the rationale for behavioral and pharmaceutical interventions to reduce symptom burden [6]. Since then, there has been substantial progress toward understanding the mechanisms and predictors that underlie post-treatment symptom burden.

Researchers have perhaps made the most progress in the area of cancer-related fatigue. The National Comprehensive Cancer Network (NCCN) defines cancer-related fatigue as an unusual, persistent, subjective sense of tiredness related to cancer or cancer treatment that interferes with usual functioning [7]. Once thought to be a byproduct of depressive symptoms, it is now a widely accepted condition with distinct features that differ from depression. Indeed, depressive symptoms and disturbed sleep are the strongest predictors of fatigue, however it has become clear that fatigue is not explained solely by depression or poor sleep [8]. Depression, sleep disturbance, and fatigue are disparate such that the cognitive and emotional characteristics of depression represent important distinguishing features [9]. Fatigue is the most common complaint among long-term cancer survivors [2,3], and interferes most with daily life [6,10]. Fatigue is a normal and expected response to chemotherapy and radiation [11], however, fatigue persists many years beyond cancer treatment in a substantial number of cancer survivors [12,13]. Indeed, a large-scale, longitudinal study of 763 breast cancer survivors found that 34% of the women were fatigued 5–10 years after diagnosis, compared to 35% 1–5 years after diagnosis; 21% of the women were fatigued at both assessments, suggesting cancer-related fatigue is both severe and persistent [2].

In addition to fatigue, depression is also a significant problem for breast cancer survivors. Cancer patients are four times more likely to have major depression [14]. The inherent difficulty in differentiating depressive symptoms from normal adjustment after breast cancer makes diagnosis difficult. Depression is characterized by feelings of sadness, hopelessness, helplessness, anhedonia, as well as insomnia and fatigue [15]. Depressive symptoms are high among breast cancer patients [16]. After completion of cancer treatment, depression is still a major concern. Adults with a history of cancer had a greater risk for depression than those who did not have a cancer history [17]. Indeed, breast cancer survivors are more likely to have mental health contacts and use mental health services than those without a cancer history [18].

Poor sleep is also a substantial problem for breast cancer survivors [19,20]. Sleep is measured by polysomnography, actigraphy, and self-report questionnaires such as the Pittsburgh Sleep Quality Index [21]. In general, these assessment tools characterize sleep quality as sleep duration, sleep latency, number of arousals, as well as subjective aspects such as feeling restful [21]. Disrupted sleep impacts between 30% and 50% of cancer patients, and persists after primary treatment in a number of breast cancer survivors [19,20]. As in other populations, sleep disturbances are associated with anxiety and depressive symptoms [19,20].

Behavioral symptoms such as fatigue, depression, and sleep disturbance are associated with substantial impairment in quality of life and also contribute to disease related outcomes. Although independently assessed, these symptoms often cluster [22]. In addition to assessing symptom severity, the construct of symptom interference is frequently utilized by the survivorship community. Symptom interference is essentially the degree to which symptoms interfere with the major aspects of a patient’s daily life [23]. The combined effects of all symptoms (often referred to as symptom burden) relative to a patient’s ability to function as he or she did before the onset of the disease or therapy is a useful metric to understand how much post-treatment symptoms impair a breast cancer survivor’s quality of life [6].

## 3. Biological Mechanisms

### 3.1. Inflammation, Sickness Behaviors, and Symptom Burden

Based on similarities between post-treatment cancer-related symptoms and cytokine-induced sickness behavior in animal models, researchers have devoted considerable work to the notion that post-treatment symptoms in cancer survivors may have an inflammatory basis [24]. Animal studies on neural-immune signaling have demonstrated that proinflammatory cytokines signal the central nervous system to trigger a constellation of behavioral changes that include fatigue, sleep disturbance, and depressive-like symptoms. Indeed, sickness behavior responses are observed in animals after the administration of infectious or inflammatory agents or certain proinflammatory cytokines [25]. Physically ill humans and animals exhibit sickness behaviors when exposed to infections. These behaviors are adaptive in that they help sick people restructure their perceptions and actions in order to conserve energy and resources. As described below, several cancer-related symptoms may be a partial side effect of persistent low-grade inflammation, representing a maladaptive version of inflammatory induced sickness behaviors [26].

Inflammation has been found to be a key mechanism underlying the symptom cluster of fatigue, depression, and sleep disturbance [26]. Fatigued breast cancer survivors had higher levels of proinflammatory activity including interleukin-1 receptor antagonist (IL-1ra), soluble tumor necrosis factor receptor Type II (sTNF-RII), and neopterin (a marker of macrophage activation), than breast cancer survivors who were not fatigued [10]. In another study, fatigued survivors had higher levels of soluble markers of proinflammatory cytokines IL-1ra and soluble interleukin-6 receptor (sIL-6r) than non-fatigued survivors [27]. Similarly, *ex vivo* production of interleukin-6 (IL-6) and tumor necrosis factor-alpha (TNF-α) by lipopolysaccharide (LPS) stimulated monocytes was higher among fatigued compared to non-fatigued breast cancer survivors [28]. Finally, fatigued breast cancer survivors had greater increased LPS-stimulated IL-1β (beta) and IL-6 production from baseline to 30 min after the Trier Social Stress Task (a standardized laboratory stressor) than non-fatigued survivors [29].

Inflammation not only induces symptoms of sickness and fatigue, but also major depressive disorder [30,31,32,33,34,35]. Indeed, both syndromal depression as well as higher levels of depressive symptoms has been linked to heightened inflammation [30,31,32,33,34,35]. Even mild depressive symptoms have been linked to elevated proinflammatory cytokine production [31,32,36]. Depression can also promote inflammation creating a problematic feedback loop [37]. Psychological stressors can provoke transient increases in proinflammatory cytokines [30,31,32,33,34,35,38,39,40,41,42,43,44,45,46,47]. Evidence from animal and human studies suggests that stress and depression can permanently alter the responsiveness of the immune system; stressors can prime the inflammatory response promoting larger cytokine increases in response to subsequent stressors and/or minor infectious challenges [34,41,48,49,50,51]. Thus, it is not surprising that chronic stressors have been linked to sustained overproduction of a key proinflammatory cytokine, IL-6 [46].

Recent work has highlighted that inflammation is also associated with sleep disturbances. Indeed, inflammation is common among individuals with sleep disorders, as well as among those with objectively assessed sleep disturbance [52,53,54,55,56,57]. Between 40% and 50% of breast cancer survivors report sleep problems that appear to be partially attributable to elevations in inflammation [22]. Although inflammation is common among individuals with sleep disorders that are objectively measured, the relationship between self-reported sleep problems and inflammation is tenuous [22].

### 3.2. Stress and Inflammation: A Pathway to Symptom Burden

As reviewed elsewhere, there are multiple different factors that likely contribute to inflammatory induced symptoms in breast cancer survivors [58]. A primary tenant of the biobehavioral model that guides our research is that stress modulates autonomic and neuroendocrine discharge, which in turn, ultimately dysregulates inflammatory activity. We propose that high levels of stress directly promote symptom burden through autonomic, neuroendocrine, and immune dysregulation.

Both physical and psychological stressors can directly provoke increases in proinflammatory cytokines [30,31,32,33,34,35,38,42,43,44,45,46,47]. Furthermore, stress and depression also contribute to greater risk for infection, prolonged infectious episodes, and delayed wound healing [59,60,61,62,63], all processes that indirectly fuel sustained proinflammatory cytokine production. Compounding these risks, poor sleep, one very commonplace consequence of stress and depression, enhances inflammation [54,55,56].

There is evidence that greater stress or distress is associated with greater immune dysregulation in both cancer [64,65,66,67,68,69,70], and non-cancer populations [31,71,72,73,74,75,76]. Elevations in inflammatory markers can also be induced experimentally, using a standardized experimental performance task, such as the Trier Social Stress Test (TSST) [77,78,79]. This allows for the observation of individual differences in acute stress reactivity under controlled experimental conditions. Breast cancer survivors who are more fatigued show a greater inflammatory stress response than those who are not fatigued. Indeed, in a sample of 10 fatigued breast cancer survivors and 15 non-fatigued breast cancer survivors, those who were fatigued showed greater cytokine production when exposed to an experimental stressor compared to those who were not fatigued [80]. In a non-cancer sample, a laboratory stressor led to higher IL-6 responses in those with more depressive symptoms, compared with those experiencing less depressive symptoms [77].

Dysregulation of the hypothalamic-pituitary-adrenal (HPA) axis, and particularly the stress hormone cortisol, has been implicated in the etiology of depression, and to a lesser extent fatigue and sleep in a large body of animal and human research. A healthy cortisol pattern includes a peak early in the morning and then decreases throughout the day [10]. In one study, fatigued breast cancer survivors had lower levels of morning serum cortisol than non-fatigued controls [10]. In another study, fatigued breast cancer survivors had flatter cortisol slopes across the day than non-fatigued survivors, as well as a rapid decline in cortisol levels in the evening [81]. Although cortisol generally inhibits inflammation, the persistence of high cortisol levels can lead immune cells to decrease their response to cortisol. Once a cell has become insensitive to glucocorticoids like cortisol, proinflammatory cytokines are produced in an unregulated environment. In turn, this environment *enhances* inflammation [82].

The autonomic stress response enhances sympathetic activity, which drives the “fight or flight response.” It also typically dampens parasympathetic activity. Because the autonomic nervous system can directly innervate immune organs, this autonomic profile of higher sympathetic and lower parasympathetic activity can raise inflammation [2,3,29,83,84,85]. Mechanistically, norepinephrine-dependent adrenergic stimulation activates nuclear factor kappa B (NF-κB), and NF-κB activates gene expression and production of proinflammatory cytokines associated with cancer-related fatigue [86]. Furthermore, lower parasympathetic activity results in higher levels of inflammation via the cholinergic anti-inflammatory pathway that facilitates acetylcholine release [87]. When acetylcholine interacts with the macrophage’s alpha-7 nicotinic receptor, it inhibits proinflammatory cytokine production [87]. Fatigued breast cancer survivors had higher sympathetic activity (as indexed by the catecholamine, norepinephrine) and lower parasympathetic activity (as indexed by high frequency heart rate variability or HF-HRV) compared with those who were not fatigued. This study was replicated by another laboratory [3]. This profile of autonomic activity (especially low parasympathetic activity) has been linked to depression and sleep disturbance as well [88,89,90,91,92,93,94,95,96].

## 4. Psychosocial Moderators

Stress is thought to play an important role in symptom burden by dysregulating the autonomic, neuroendocrine, and immune systems [97]. Research on stress and symptom burden has typically conceptualized stress as an acute trigger for the onset of disease and associated symptoms [97]. Recently, behavioral scientists have taken a life-course perspective for the study of stress and disease [98]. This perspective highlights that cumulative exposure to various types of adversity has a profound impact on symptom burden by dysregulating the autonomic, neuroendocrine, and immune systems [98]. Accordingly, stressful life experiences such as abuse, being low socioeconomic status (SES), or being a victim of discrimination and prejudice may all impact cancer-related symptoms.

Trauma victims are particularly vulnerable to emotional distress and somatic symptoms after breast cancer treatment. Adult trauma survivors suffered more cancer-related distress than those who did not report traumatic experiences after treatment for breast cancer [99]. Further, breast cancer survivors who were abused or neglected as children reported more psychological distress, poorer functional well-being, and more cancer related fatigue compared with who did not report childhood abuse [100]. Child adversity was also associated with elevated markers of inflammation in breast cancer survivors [101]. It is possible that profound stressors such as these prime the inflammatory stress response making women particularly vulnerable to post-treatment symptoms after cancer treatment.

People of lower socioeconomic position are confronted with a number of important social and environmental conditions that contribute to chronic stress and depression, which leads to considerable cumulative stress exposure over the life course [102,103,104]. Socioeconomic disparities in cancer-related symptom burden exist such that those with lower socioeconomic status are disproportionally burdened by post-treatment cancer-related fatigue, depression, and sleep problems [2,5,10,13,27,85,105,106]. These findings echo the well-established inverse, monotonic relationship between socioeconomic status and other health outcomes [102].

Ethnic minority breast cancer survivors report significantly worse symptom burden than others [107]. These disparities are thought to arise from a combination of economic, social, and cultural factors. Among breast cancer survivors, African American and Hispanic survivors report significantly poorer physical functioning compared to Caucasian and Asian American survivors [108]. Among breast cancer survivors, African American and Hispanic survivors report significantly lower physical functioning compared to Caucasian and Asian American survivors [108]. The US Latino population is growing rapidly, and Mexican-Americans form the largest Latino subgroup. On average, Latinos have very low socioeconomic status [109]. It is possible that the effects of Latino ethnicity and low socioeconomic status are interactive. Specifically, the effects of low socioeconomic status may be heightened in Latinos due to exposure to stressors specific to being a minority.

Social support can serve as a protective factor for reducing cancer-related symptoms [110]. Although social support can be defined in many ways, it generally is the perception or experience that you are loved and cared for by others, esteemed and valued, and part of a social network [111]. Higher levels of social support predicted improvements in subjective well-being over time among breast cancer survivors [112]. Breast cancer patients who reported greater life stress also experienced less mood disturbances if they had more people in their support network [113]. The beneficial effects of social support seem to exist cross-culturally. In Taiwan, social support buffered breast cancer survivors’ depressive symptoms [114]. In other work, social support predicted the degree to which breast cancer survivors reported positive personal growth as a result of their cancer [112].

## 5. Exercise to Improve Symptom Burden

Exercise may be one of the most important health behaviors for cancer survivors to maintain. Physical activity level is related to symptom burden [115]. In one study, survivors who met the general physical activity recommendation had less symptom burden than those who did not [116]. As evidenced in multiple intervention studies, exercise may even be powerful enough to change cancer survivors’ post-treatment symptoms [117]. This seems to be true even among ethnic minorities. For example, an exercise intervention improved physical fitness, reduced perceived stress, and decreased cortisol levels among Hispanics [118]. Although intense exercise regiments are not always possible for breast cancer survivors, moderate exercise regiments, such as yoga, may be particularly helpful. Indeed, yoga improved mood, alleviated fatigue, and lowered inflammation in breast cancer survivors [119].

## 6. Pharmaceutical Treatments to Improve QOL

Several barriers have hindered the development of clinical trials in symptom management. First, the subjective nature of fatigue symptoms has limited innovative research into the mechanisms underlying these symptoms and the development of novel ways of treating or preventing them. However, patient-reported outcome research has recently been promoted by the U.S. Food and Drug Administration (FDA) for more accurate therapeutic agent evaluation, and symptom reduction has been recognized as a primary clinical benefit for drug approval [120,121]. In addition, until recently, there has been a lack of understanding of the biological mechanisms that underlie subjective symptoms.

Perhaps the most obvious drug to treat these post-treatment symptoms in breast cancer survivors is selective serotonin reuptake inhibitors (SSRIs). Indeed, newer antidepressants are widely used in women with breast cancer for treatment of depression and are prescribed for tamoxifen related hot flashes and various other indications. Although SSRIs are useful in the treatment of depression in this population, they do not appear to impact fatigue or pain [122].

The control or prevention of cancer-related cytokine dysregulation presents a novel opportunity for symptom reduction. However, many of the agents that might be effective in the control of inflammation are generic, and sometimes even over the counter. Nonsteroidal anti-inflammatory drugs (NSAIDs) are one such drug. NSAIDs inhibit proinflammatory cytokines by inhibiting the enzyme cyclooxygenase (Cox), which is responsible for inflammatory production. Different NSAIDs inhibit the activity of either cyclooxygenase-1 (COX-1) or cyclooxygenase-2 (COX-2), or both to different degrees, and thereby, the synthesis of prostaglandins and thromboxanes. NSAIDs are weak acids that are well absorbed from the stomach and intestinal mucosa where they are then metabolized in the liver.

Studies have investigated whether the use of anti-inflammatory agents improve the antidepressant response for treatment resistant depression where inflammation is the likely culprit. A recent meta-analysis of 10 trials (4258 participants) revealed that anti-inflammatory treatment reduces depressive symptoms compared with placebo [123]. Across 10 trials, NSAIDS reduced depressive symptoms without adverse effects. Importantly, the antidepressant effect was shown to be independent of pain relief. There have been no studies investigating the direct effect of NSAIDS on cancer-related symptoms; this is an interesting avenue for future research [123].

## 7. Conclusions

Being diagnosed and treated for breast cancer is emotionally and physically challenging. Even when treatment-related problems subside, many breast cancer survivors report symptoms of depression, fatigue, and sleep disturbance [5]. The physical and psychological effects of the aftermath of a cancer diagnosis and its treatment are notable. Psychological stress substantially augments adverse autonomic, endocrine, and immune discharge, including enhanced production of proinflammatory cytokines [124]. Importantly, inflammation is a key biological mechanism underlying the symptom cluster of depression, fatigue, and sleep disturbances; there is also good evidence that inflammation contributes to breast cancer recurrence [6,125]. Stress may exert direct effects on psychological and physiological risk processes [98,126]. As the population of breast cancer survivors grows, a biobehavioral approach to research and treatment will be needed.
